# Supplementary material for: Simultaneous Down-Regulation of Intracellular MicroRNA-21 and hTERT mRNA Using AS1411-Functionallized Gold Nanoprobes to Achieve Targeted Anti-Tumor Therapy
Source: Nanomaterials (Basel). 2024 Dec 5;14(23):1956. doi: 10.3390/nano14231956 (PMC11643209; doi:10.3390/nano14231956)
Supplement: Supplementary file 1 [file nanomaterials-14-01956-s001.zip › nanomaterials-3323273-supplementary.pdf]

Electronic supporting information

Simultaneous down-regulation of intracellular microRNA-21 and hTERT mRNA using AS1411-functionallized gold nanoprobes to achieve targeted anti-tumor therapy

Qinghong Ji, Qiangqiang Yang, Mengyao Ou and Min Hong*

School of Chemistry and Chemical Engineering, Liaocheng University, Liaocheng, 252059, China

17861826032@163.com (Q.J.); mortalyang@163.com (Q.Y.); 15275820795@163.com (M.O.)
* Correspondence: hongmin@lcu.edu.cn

1. Reagents

Chloroauric acid ($HAuCl_4·4H_2O$), potassium dihydrogen phosphate (KH_2PO_4), disodium hydrogen phosphate (Na_2HPO_4), sodium citrate ($C_6H_5Na_3O_7$), sodium chloride (NaCl) and potassium chloride (KCl) were purchased from Shanghai Chemical Reagent Company (China). RNase inhibitor (DEPC), 3-(4,5-dimethylthiazole-2)-2,5-diphenyltetrazolium bromide salt (MTT) and Tween-20 were purchased from Sigma-Aldrich Co., Ltd (USA). All other reagents are analytical grade. Phosphate buffer (PBS, 136.7 mM NaCl, 2.7 mM KCl, 8.72 mM Na_2HPO_4, 1.41 mM KH_2PO_4, pH=7.4). FITC-Annexin V, PI Apoptosisi Kit and the total RNA extraction kit were

*Corresponding author. Tel.: +866358239195; Fax: +866358239121.
E-mail address: hongmin@lcu.edu.cn (M. Hong).

purchased from Shanghai Beyotime Biotech. Co., Ltd. Oligonucleotides used in this work (Table S1) were purchased from Sangon Biotech (Shanghai) Co., Ltd.

Table S1. DNA sequences used in this work.

Name	Sequences
HS-anti-hTERT-DNA	5'-TCCATGTTCACAATCGGCCA$_{10}$-SH-3'
Cy3-hTERT-DNA	5'-Cy3-GCCGATTGTGA-3'
hTERT-DNA	5'-GCCGATTGTGA-3'
Target-hTERT-DNA	5'-GGTCGATTGTGAACATGGA-3'
HS-miRNA-21-DNA	5'-HS-A$_{10}$ TAGCTTATCAGACTGA-3'
Cy5-AS1411-anti-miRNA-21-DNA	5'-(GGT)$_4$ TGT(GGT)$_3$ GG -S-S-TCAACATCAGTC TGATAAGCT-Cy5-3'
AS1411-anti-miRNA-21-DNA	5'-(GGT)$_4$ TGT(GGT)$_3$ GG -S-S-TCAACATCAGTC TGATAAGCT-3'
Target-miRNA-21-DNA	5'-TAGCTTATCAGACTGATGTTGA-3'
HS-Control-DNA	5'-HS-A$_{10}$ CAGTCTAACTGAATTG-3'
Control-DNA	5'-TCAACACAATTCAGTTAGACTG-3'
hTERT forward primer	5'-CGGAAGAGTGTCTGGAGCAA-3'
hTERT reverse primer	5'-CACGACGTAGTCCATGTTCA-3'
GAPDH forward primer	5'-CTCAGACACCATGGGGAAGGTGA-3'
GAPDH reverse primer	5'-ATGATCTTGAGGCTGTTGTCATA-3'

2. Cells and animals

Two cancer cell lines, including HeLa (human cervical cancer cells) and MCF-7 (human breast cancer cells), were purchased from the American Type Culture Collection (ATCC) and cultured in DMEM with 10% (vol/vol) fetal bovine serum and 1% (vol/vol) penicillin/streptomycin mixture at 37 °C with 5 % CO_2.

Balb/c female nude mice (3~4 weeks old) were purchased from Pengyue Laboratory Animal Center (Jinan). The animal study was in compliance with the National Guide for Care and Use of Laboratory Animals and the experimental protocols were approved by the Special Committee of Scientific Research Ethic of Liaocheng University) (No. 2023022701).

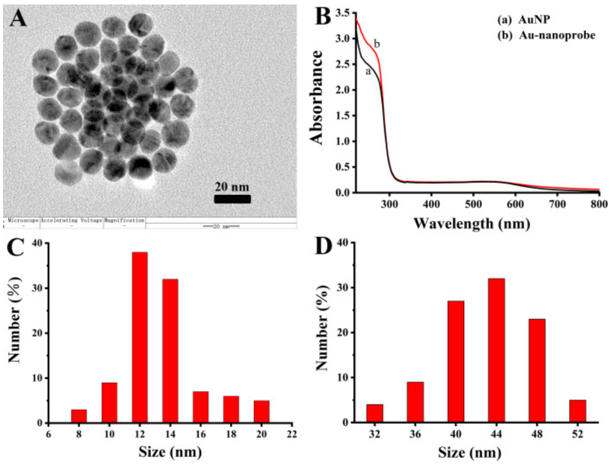

Figure S1. (A) TEM characterization of AuNPs. (B) UV-Vis absorption spectra of AuNPs and Au-nanoprobes. Hydrodynamic size distribution of AuNPs (C) and Au-nanoprobes (D) determined by DLS.

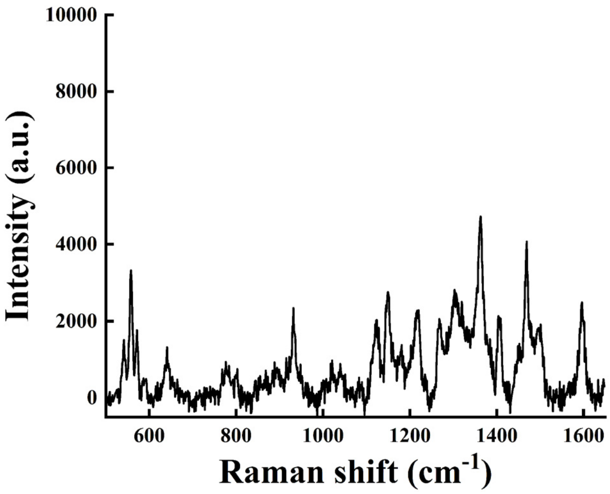

Figure S2. Surface enhanced raman spectrum of Au-nanoprobes.

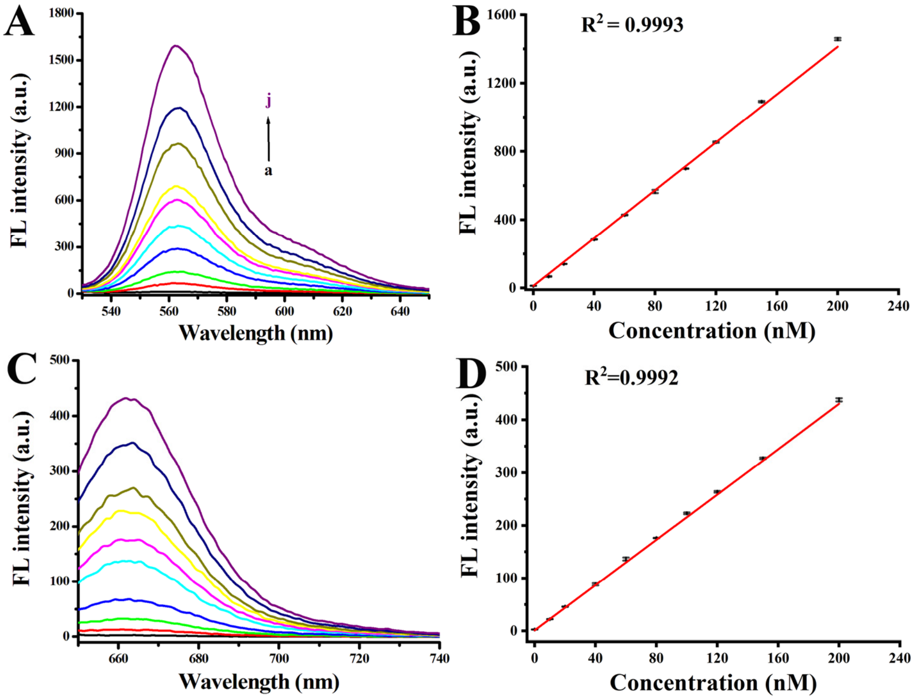

Figure S3. The fluorescence spectra of HS-anti-hTERT-DNA/Cy3-hTERT-DNA (A) or HS-miRNA-21-DNA/Cy5-AS1411-anti- miRNA-21 duplexes (C) with the concentrations of 0, 10, 20, 40, 60, 80, 100, 120, 150, and 200 nM. The relationship between the fluorescence intensity and different concentrations of HS-anti-hTERT-DNA/Cy3-hTERT-DNA (B) or HS-miRNA-21-DNA/Cy5-AS1411-anti- miRNA-21 duplexes (D). Error bars show the standard deviations of three experiments.
